# Transcriptional Regulation of Thrombin-Induced Endothelial VEGF Induction and Proangiogenic Response

**DOI:** 10.3390/cells10040910

**Published:** 2021-04-15

**Authors:** Rusan Catar, Guido Moll, Isa Hosp, Michele Simon, Christian Luecht, Hongfan Zhao, Dashan Wu, Lei Chen, Julian Kamhieh-Milz, Katarzyna Korybalska, Daniel Zickler, Janusz Witowski

**Affiliations:** 1 Department of Nephrology and Internal Intensive Care Medicine, Charité—Universitätsmedizin Berlin, Corporate Member of Freie Universität Berlin and Humboldt-Universität zu Berlin, 10117 Berlin, Germany; isa@lutzschramm.de (I.H.); michele.simon@charite.de (M.S.); christian.luecht@charite.de (C.L.); hongfan.zhao@charite.de (H.Z.); Dashan.wu@charite.de (D.W.); lei.chen1991@gmail.com (L.C.); daniel.zickler@charite.de (D.Z.); jwitow@ump.edu.pl (J.W.); 2 BIH Center for Regenerative Therapies (BCRT) and Berlin-Brandenburg School of Regenerative Therapies (BSRT), Berlin Institute of Health (BIH), Charité—Universitätsmedizin Berlin, 10117 Berlin, Germany; 3 Institute of Transfusion Medicine, Charité—Universitätsmedizin Berlin, 10117 Berlin, Germany; julian.milz@charite.de; 4 Department of Pathophysiology, Poznan University of Medical Sciences, 60-806 Poznan, Poland; koryb@ump.edu.pl

**Keywords:** cardiovascular diseases (CVD), endothelial cells (ECs), innate immune response, thromboinflammation, thrombin, protease-activated receptor 1 (PAR-1), activator protein 1 (AP-1) transcription factor complex, vascular endothelial growth factor (VEGF), angiogenesis

## Abstract

Thrombin, the ligand of the protease-activated receptor 1 (PAR1), is a well-known stimulator of proangiogenic responses in vascular endothelial cells (ECs), which are mediated through the induction of vascular endothelial growth factor (VEGF). However, the transcriptional events underlying this thrombin-induced VEGF induction and angiogenic response are less well understood at present. As reported here, we conducted detailed promotor activation and signal transduction pathway studies in human microvascular ECs, to decipher the transcription factors and the intracellular signaling events underlying the thrombin and PAR-1-induced endothelial VEGF induction. We found that c-FOS is a key transcription factor controlling thrombin-induced EC VEGF synthesis and angiogenesis. Upon the binding and internalization of its G-protein-coupled PAR-1 receptor, thrombin triggers ERK1/2 signaling and activation of the nuclear AP-1/c-FOS transcription factor complex, which then leads to *VEGF* transcription, extracellular secretion, and concomitant proangiogenic responses of ECs. In conclusion, exposure of human microvascular ECs to thrombin triggers signaling through the PAR-1–ERK1/2–AP-1/c-FOS axis to control *VEGF* gene transcription and VEGF-induced angiogenesis. These observations offer a greater understanding of endothelial responses to thromboinflammation, which may help to interpret the results of clinical trials tackling the conditions associated with endothelial injury and thrombosis.

## 1. Introduction

**Figure 1:**
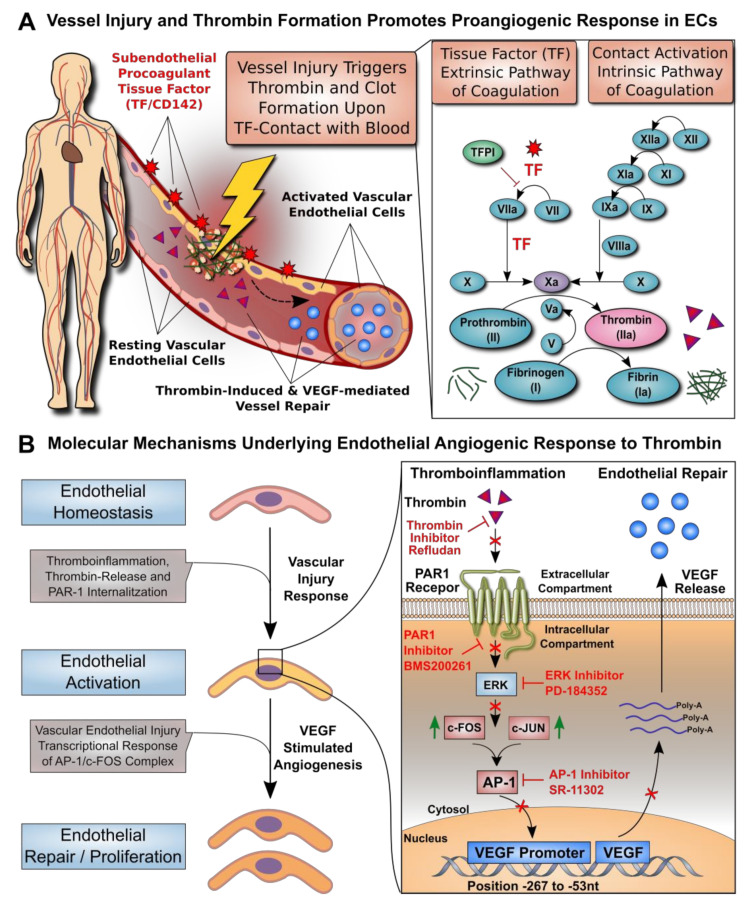
Transcriptional regulation of thrombin-induced endothelial cell VEGF induction. (**A**) Vessel injury and thrombin formation promote a proangiogenic response in ECs. While resting vascular ECs form a protective anticoagulant lining that prevents uncontrolled blood activation at the blood–endothelial interface [8], different types of pathological conditions can promote activation of vascular ECs, leading to thrombin and clot formation. Thromboinflammation may be triggered upon disruption of the protective endothelial interface, thereby allowing the detrimental contact of blood with subendothelial highly procoagulant tissue factor (TF/CD142), which can induce the extrinsic TF pathway of coagulation. In addition, the intrinsic contact activation pathway of coagulation may be activated through multiple triggers. Activation of either coagulation pathway leads to conversion of prothrombin to thrombin and fibrinogen to fibrin, with thrombin being able to stimulate EC activation and release of VEGF with concomitant EC proliferation and repair. (**B**) Molecular mechanisms underlying endothelial angiogenic response to thrombin. ECs can be activated by various stimuli (e.g., vascular injury, thromboinflammation, and thrombin release), which can promote EC proliferation and repair (e.g., VEGF-stimulated angiogenesis). We here identified that these events are mediated through a signaling cascade involving PAR-1, ERK, and AP-1 signaling, with concomitant VEGF promoter engagement (Position −267 to −53 nucleotides from the *VEGF* gene), VEGF mRNA synthesis, and VEGF protein secretion.

Cardiovascular diseases (CVDs) are a leading cause of death worldwide [1,2]. Endothelial cell (EC) dysfunction is a critical stage in the development of many CVDs [3]. The endothelium provides a thromboresistant inner lining of the vascular wall, thus preventing uncontrolled clot formation [3,4]. Several acute and chronic pathologies can promote EC dysfunction and predispose the interface to a prothrombotic state [5,6]. The inflammation that is associated with these conditions can promote EC activation and expression of highly procoagulant tissue factor (TF/CD142) [7,8,9], thereby promoting thromboinflammation and the generation of thrombin (Figure 1A). The generation of thrombin in the vicinity of ECs leads to endothelial activation and the release of vascular endothelial growth factor (VEGF) to stimulate endothelial repair.

Under physiological conditions, thrombin acts on ECs through protease-activated receptor-1 (PAR-1) to counteract excessive thrombotic responses. Thrombin can stimulate protective responses in ECs to release nitric oxide, prostacyclin, and tissue-type plasminogen activator, which cause vasodilation, platelet inhibition, and fibrinolysis, respectively [4,10]. Thrombin also contributes to wound healing by promoting angiogenesis, vascular remodeling, and antimicrobial activity [11,12,13]. These thrombin activities are mediated through intricate transcriptional networks, whose operations have only been partially deciphered so far [3,4]. The effect of thrombin on dysfunctional endothelium is less well understood. Presumably, during the induction and progression of endothelial dysfunction, thrombin may trigger aberrant responses that cause further deterioration, thus adversely affecting vascular tone, permeability, and angiogenesis.

Angiogenesis can be modulated by thrombin through different mechanisms [14], including direct effects on the endothelium, such as potent stimulation of EC proliferation and migration (Figure 1B, left panel) [15,16,17], but also through indirect mechanisms, such as upregulation of the receptors responsive to angiogenic stimuli and increased expression of adhesion receptors and integrins for EC migration and survival [18,19,20]. Thrombin can amplify the effects of VEGF by increasing the expression of functional VEGF receptors on ECs [20,21]. Increased expression of the *VEGF* gene in response to thrombin was detected in several cell types, including platelets [22,23,24], fibroblasts [22], astrocytes [24], retinal pigment epithelial cells [25,26,27], vascular smooth muscle cells [28,29], adipocytes [30], and mesothelial cells [31], as well as several malignant cell types [22,32,33,34,35]. A first study examining this aspect in human ECs documented thrombin-induced *VEGF* expression in human umbilical vein ECs and suggested the involvement of hypoxia-induced transcription factor 1 alpha [36]. Intriguingly, thrombin is known to induce many transcription factors in ECs [11], and the *VEGF* promoter contains numerous putative binding sites for transcription factors [37]. 

The connection between thrombin and VEGF has received renewed interest during the COVID-19 pandemic, due to the pronounced coagulopathy in severely affected patients [38], which develop acute respiratory distress syndrome (ARDS), vascular leakage and altered angiogenesis [39]. VEGF is a key mediator of pathological angiogenesis [40] and it has also long been suspected to contribute to the pathogenesis of ARDS [41]. To date, a detailed analysis of the transcriptional regulation of *VEGF* expression by thrombin in ECs and its link to endothelial angiogenesis has been lacking. We here show that the activator protein 1 (AP-1)/c-FOS transcription complex is a key regulator of the VEGF-mediated angiogenic response of ECs to thrombin (Figure 1B, right panel).

## 2. Materials and Methods

### 2.1. Materials

Unless stated otherwise, all chemicals were from Sigma-Aldrich (St Louis, MO, USA), and all culture plastics were from Becton Dickinson (Falcon; Franklin Lakes, NJ, USA). Cell culture media and buffers were from Thermo Fisher (Waltham, MA, USA) and fetal calf serum (FCS) from Invitrogen (Darmstadt, Germany). The antibody characteristics are given in the Supplementary Materials (Tables S1 and S2). Thrombin used was α-thrombin from human plasma with concentrations reported in NIH units of activity.

### 2.2. Endothelial Cell Culture, Tube Formation Assay, and VEGF Protein and Gene Expression

Human microvascular endothelial cells (HMECs, catalogue no. CRL-3243) were purchased from ATCC^®^ (Manassas, VA, USA) and used at passage 2–6. The EC tube formation assay was conducted as outline previously [42,43]. Briefly, Matrigel (Corning, Tewksbury, MA, USA) was poured onto a 96-well plate (50 µL/well) and solidified at 37 °C for 30 min. HMECs were suspended in MCDB131 culture medium supplemented with penicillin, streptomycin, glutamine (all at standard cell culture doses), hydrocortisone (10 nM), and 0.5% FCS, and then seeded onto the Matrigel (2 × 10^4^ cells/well) and stimulated as specified in figure legends. The formation of capillary tube networks was evaluated after 16 h and photographed under the microscope (Zeiss Axiovert 40 CFL Oberkochen, Germany), and three randomly selected fields were analyzed for different parameters of endothelial tube network formation (e.g., the number of meshes) using ImageJ 1.43 software (National Institutes of Health, USA). VEGF protein was measured using a DuoSet Immunoassay Kit (R&D Systems, Minneapolis, MN, USA) [44]. Expression of the *VEGF* gene (and *β2M* as a housekeeping gene) was assessed with reverse transcription and quantitative PCR (RT-qPCR), as described previously [42,45,46,47,48].

### 2.3. DNA Construct, Transient Transfection, and Analysis of the VEGF Promoter

The DNA constructs of predefined VEGF promoter fragments were provided by Dr. A. Scholz [49] and checked for correct length of promoter segments by restriction digest. For transient transfection studies, cells were seeded into 6-well culture plates and transfections performed at 70–80% cell confluence in the absence of serum using the TurboFect™ transfection reagent (Thermo Fisher). The HMECs were transfected with the VEGF reporter plasmids and the reference plasmids and assayed with the dual-luciferase reporter assay system (Promega) [35]. The human *VEGF* promoter region located at −268 to −51 nt (GenBank NT_007592.15) was analyzed with the PROMO virtual laboratory for the presence and location of potential transcription factor binding sites: http://alggen.lsi.upc.es/cgi-bin/promo_v3/promo/promoinit.cgi?dirDB=TF_8.3, accessed on 11 April 2021.

### 2.4. Nuclear Extracts and Electrophoretic Mobility Shift Assay

Nuclear extracts were prepared using the NE-PER Nuclear and Cytoplasmic Extraction Kit and oligonucleotide probes labeled with Biotin 3’ End DNA Labeling Kit (all Thermo Fisher). For the electrophoretic mobility shift assay (EMSA) [42], the following probes were used (corresponding region of the *VEGF* promoter given in brackets, Figure S1D): c-FOS 5′-CTGAGCGTCCGCAGAGCCCGGGCC-3′ (−131 to −154) and c-JUN 5′-GTAATTTTCAGGCTGTGAACCTTG-3′ (−205 to −228). Each binding mixture (20 µL) contained 5 µg nuclear extract, 20 fmol labeled double-stranded probe, 1 µg poly-dI/dC, and 2 µL 10× buffer and was incubated at room temperature for 30 min. Protein–DNA complexes were analyzed by electrophoresis in 6% nondenaturing polyacrylamide gels and visualized with LightShift Chemiluminescent EMSA Kit (Thermo Fisher).

### 2.5. Western Blotting Assays and Flow Cytometry on PAR-1 Expression and Internalization

The cell extracts were prepared as described [48], electrophoresed on sodium dodecyl sulfate–polyacrylamide gels, and Western blotted using antibodies against c-FOS and c-JUN (Santa Cruz Biotechnology), pERK-1/2, and β-actin (Cell Signaling Technology, Frankfurt, Germany), and appropriate secondary peroxidase-conjugated IgG (Dianova, Hamburg, Germany). The bands obtained were analyzed using the Enhanced Chemiluminescence Detection System (Thermo Scientific) and ImageJ 1.43 software. PAR1 receptor expression and cleavage was assessed with flow cytometry (FACS Aria; Becton Dickinson). Activation of PAR-1 was monitored with the SPAN12 monoclonal antibody detecting a PAR-1 epitope that only exists in the uncleaved receptor, with loss of SPAN12 staining pointing to PAR-1 activation [50]. All experimental steps were performed at 4 °C to prevent unspecific PAR-1 activation and internalization. HMECs were diluted (3:7) in PBS and incubated for 10 min with either SPAN12 antibody or a buffer, and then fixed with 1% paraformaldehyde and analyzed with flow cytometry.

### 2.6. Statistics

Statistical analysis was performed using GraphPad Prism 6.05 software (GraphPad Software). The data were analyzed with the t-test or repeated measures analysis of variance. Results were expressed as means ± SEM. Differences with a *p* value < 0.05 were considered significant.

## 3. Results

The study background and a summary of the molecular mechanisms identified in this study can be found in Figure 1. In brief, vessel injury and thrombin formation are known to promote proangiogenic responses in ECs. We here elucidate the detailed molecular mechanisms underlying the EC angiogenic response to thrombin.

### 3.1. Dose–Response Effect of Thrombin on EC Angiogenesis and VEGF Production

**Figure 2:**
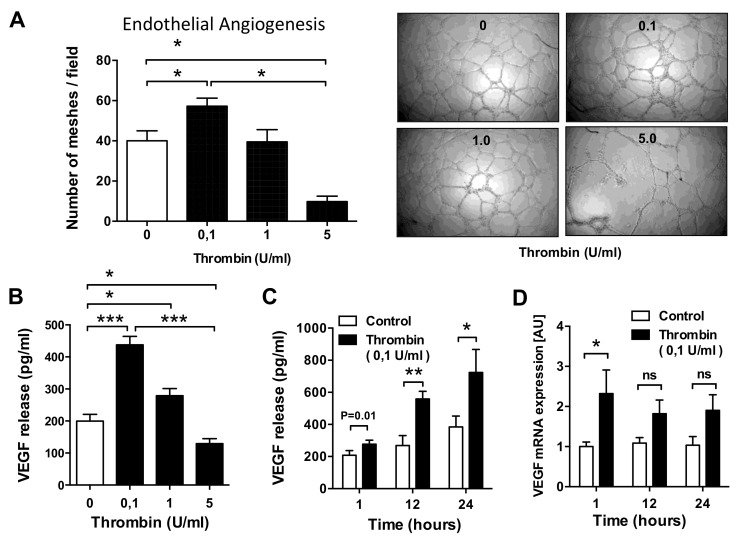
Effect of thrombin on microvascular EC angiogenesis and VEGF production. (**A**) Human microvascular endothelial cells (HMECs) were stimulated with thrombin at the indicated doses (0.1, 1.0, and 5.0 U/mL), and EC tube formation was assessed after 16 h, with data expressed as the average number of meshes per field (*n* = 4) and representative figures shown to the right. (**B**–**D**) HMECs were cultured in the presence or absence of thrombin (*n* = 8) and assessed for dose- (0.1, 1.0, and 5.0 U/mL) and time-dependent (6, 12, and 24 h) VEGF secretion (**B**,**C**) and *VEGF* mRNA levels (**C**). The exposure time in (**A**) was 24 h, while the dose of thrombin in (**C**,**D**) was 0.1 U/mL. ANOVA mean +/− SEM with * *p* < 0.05, ** *p* < 0.01, and *** *p* < 0.001.

In prior studies, we employed in vitro endothelial tube formation assays to study angiogenic properties of ECs and mesenchymal stromal cells (MSCs) [42,43], and a similar setup was chosen in this study. Exposure of HMECs to thrombin concentrations of 0.1 U/mL resulted in a 43% increase in EC network formation (*p* < 0.05; Figure 2A). Higher thrombin concentrations did not produce such an effect (1 U/mL) or even impaired (5 U/mL) endothelial tube formation. The dose of 0.1 U/mL was chosen for further studies, which is in a similar range as measured under physiological conditions [46,51]. Since VEGF is a main regulator of endothelial angiogenesis, we examined the effect of thrombin on VEGF production by ECs. Indeed, the effect of thrombin on VEGF secretion mirrored that on EC angiogenesis. Exposure of HMECs to thrombin at a concentration of 0.1 U/mL, but not at higher doses, increased VEGF production by more than 2-fold (*p* < 0.001; Figure 2B). This effect became most apparent after 12 h of stimulation (*p* < 0.01, more than 2-fold increase; Figure 2C) and was preceded by an increase in *VEGF* mRNA expression, which already peaked at 1 h post stimulation (*p* < 0.05; Figure 2D).

### 3.2. Effect of Thrombin on the VEGF Promoter Activity in Microvascular Endothelial Cells

**Figure 3:**
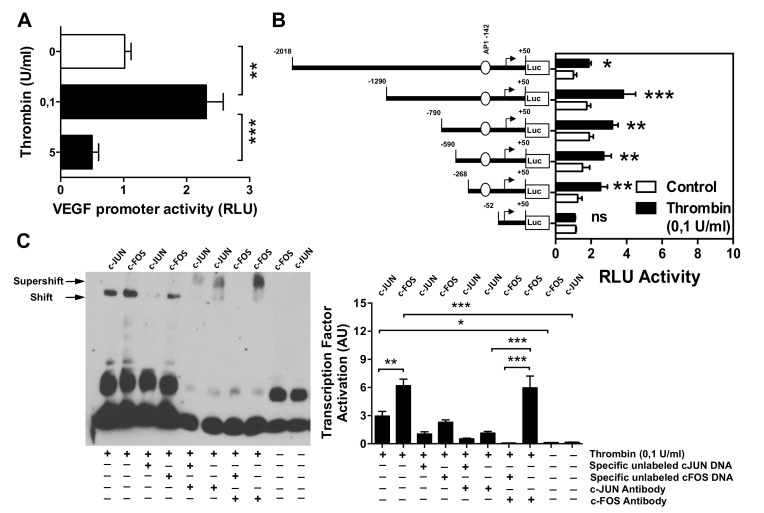
Effect of thrombin stimulation on the *VEGF* promoter. HMECs were transiently transfected with *VEGF* promoter constructs, stimulated with thrombin (0.1 U/mL) for 6 h (and 12 h, Figure S1B), and analyzed for luciferase activity. (**A**) Full-length VEGF promoter activity (*n* = 5). (**B**) Effect of progressive 5′ deletions of the *VEGF* promoter on its activity upon stimulation with thrombin (*n* = 8). (**C**) EMSA identifying the role of c-FOS and c-JUN in mediating *VEGF* promoter induction by thrombin (*n* = 4). Nuclear extracts were obtained from HMECs treated with thrombin (0.1 U/mL) for 6 h, and EMSA was performed using c-FOS and c-JUN consensus oligonucleotide probes either in the presence of an excess of specific unlabeled c-FOS or c-JUN DNA, or in the presence of c-FOS or c-JUN-specific antibodies, with quantification of shift- or supershift lanes, respectively. ANOVA mean +/− SEM with * *p* < 0.05, ** *p* < 0.01, and *** *p* < 0.001.

To investigate how thrombin affects the activity of the *VEGF* gene promoter, we employed a set of established assays [43,45,48]. The HMECs were first transiently transfected with *VEGF* luciferase reporter gene constructs and stimulated with thrombin (0.1 U/mL). This resulted in a significant increase in the full-length *VEGF* promoter activity (*p* < 0.01; Figure 3A). To identify *VEGF* promoter regions responsive to thrombin, progressive deletions of the *VEGF* promoter were performed (Figure 3B). Truncation of the promoter region spanning positions −267 to −53 abolished the ability of the VEGF promoter to respond to thrombin, suggesting that the region contained regulatory elements essential for the *VEGF* promoter activity. The *in silico* analysis pointed to the presence of high-affinity binding sites for the transcription factor c-FOS. To determine whether c-FOS mediated the effect of thrombin toward the *VEGF* promoter, an electrophoretic mobility shift assay (EMSA) was performed using a biotin-labeled consensus oligonucleotide for c-FOS or c-JUN binding that corresponded to positions −131 to −154 or −205 to −228 of the *VEGF* promoter (Figure 3C). This experiment demonstrated that nuclear extracts from cells stimulated with thrombin formed a DNA–protein complex with the c-FOS-specific oligonucleotide more efficiently than with the c-JUN oligonucleotide (*p* < 0.001 vs. *p* < 0.05). To verify the specificity of c-FOS and c-JUN binding, EMSA was performed with a 100-fold molar excess of unlabeled oligonucleotide. This competition assay resulted in a loss of c-FOS and c-JUN-DNA complex. In contrast, EMSA with a specific anti-c-FOS antibody led to a supershift of DNA–protein complex, which was much less profound for anti-c-JUN antibody (*p* < 0.001; 5 times weaker signal).

### 3.3. AP-1 Transcription Factor Mediates Thrombin-Induced EC Angiogenesis

**Figure 4:**
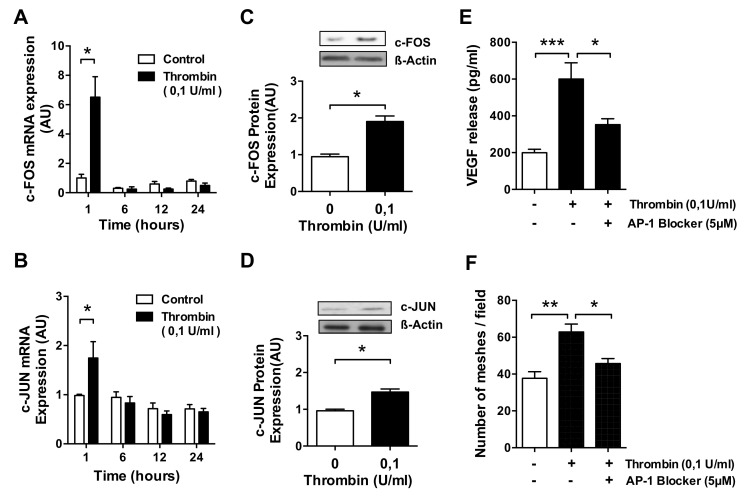
Engagement of AP-1/c-FOS/c-JUN signaling in thrombin-stimulated microvascular ECs. (**A**–**C**) HMECs were treated with thrombin (0.1 U/mL) for the times indicated and assessed for: (**A**,**B**) *c-FOS* and c-JUN mRNA level (*n* = 4); (**C**,**D**) c-FOS and c-JUN protein upregulation after 3 h of stimulation (*n* = 4); (**E**,**F**) HMECs were preincubated for 1 h with or without the AP-1 inhibitor SR-11302 (5 µM) and then stimulated with thrombin (0.1 U/mL) for 24 h, followed by the measurement of (**E**) VEGF protein release (*n* = 7–8) and (**F**) the number of endothelial network meshes formed per field (*n* = 6). ANOVA mean +/− SEM with * *p* < 0.05, ** *p* < 0.01, and *** *p* < 0.001.

Exposure of HMECs to thrombin resulted in a rapid increase in the *FOS* and *JUN* mRNA level (*p* < 0.05; Figure 4A,B), which was approximately 3-fold stronger for *FOS*, and followed by an upregulation of c-FOS and c-JUN protein (*p* < 0.05; Figure 4C–D), which was 2-fold stronger for c-FOS than c-JUN. Since both proteins—c-JUN and c-FOS—combine to form the AP-1 transcription factor complex, we assessed the effect of SR-11302, a specific AP-1 inhibitor. In HMECs pretreated with SR-11302, both the release of VEGF (*p* < 0.001 and *p* < 0.05; Figure 4E) and the formation of tubes in response to thrombin (*p* < 0.01 and *p* < 0.05; Figure 4F) were almost reduced to basal levels, with some residual VEGF release remaining, thus indicating that VEGF release is not entirely controlled by the AP-1 transcription factor complex. To verify if the concentration of the AP-1 blocking agent was optimal, we conducted a prior titration experiment, which revealed that the best blocking effect was achieved with 5 uM AP-1 blocker (Figure S1C).

### 3.4. ERK Blockade Impairs Thrombin-Induced VEGF Expression by ECs

**Figure 5:**
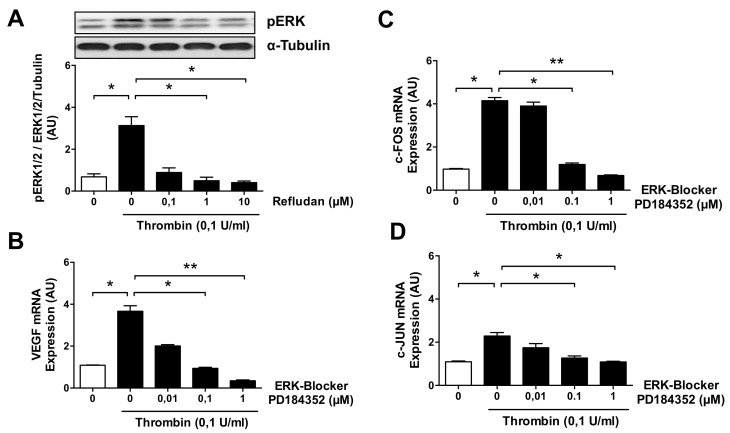
ERK1/2 and AP-1/c-FOS/c-JUN mediate thrombin stimulation in microvascular ECs. (**A**) HMECs were pretreated for 1 h with or without the thrombin inhibitor Refludan and then stimulated with thrombin (0.1 U/mL) for 15 min and assessed for the presence of phosphorylated ERK1/2 (*n* = 5). (**B**–**D**) HMECs were pretreated for 1 h with or without the ERK inhibitor PD-184352 and then stimulated with thrombin (0.1 U/mL) for 1 h, after which the levels of (**B**) VEGF mRNA and (**C**) FOS mRNA and (D) JUN mRNA were measured (*n* = 4). ANOVA mean +/− SEM with * *p* < 0.05 and ** *p* < 0.01.

Since the extracellular signal-regulated kinase 1/2 (ERK1/2) pathway has been previously linked to signaling from thrombin receptors, we analyzed whether thrombin affects the expression of phosphorylated ERK1/2 (pERK1/2) proteins in HMECs. Indeed, exposure of ECs to thrombin at a dose of 0.1 U/mL resulted in a strong 4.5-fold increase in pERK1/2 (*p* < 0.05; Figure 5A). Importantly, this effect was almost entirely abolished by preincubation of the HMECs with increasing concentrations of lepirudin (Refludan) (*p* < 0.05), a direct thrombin inhibitor. In addition, PD-184352, a specific ERK1/2 inhibitor, dose-dependently reduced the levels of both *VEGF* mRNA (*p* < 0.05 and *p* < 0.01; Figure 5B) and also *FOS* and *JUN* mRNA (*p* < 0.05 and *p* < 0.01; Figure 5C,D).

### 3.5. Thrombin-Induced PAR-1 Activation Modulates VEGF Expression by ECs

**Figure 6:**
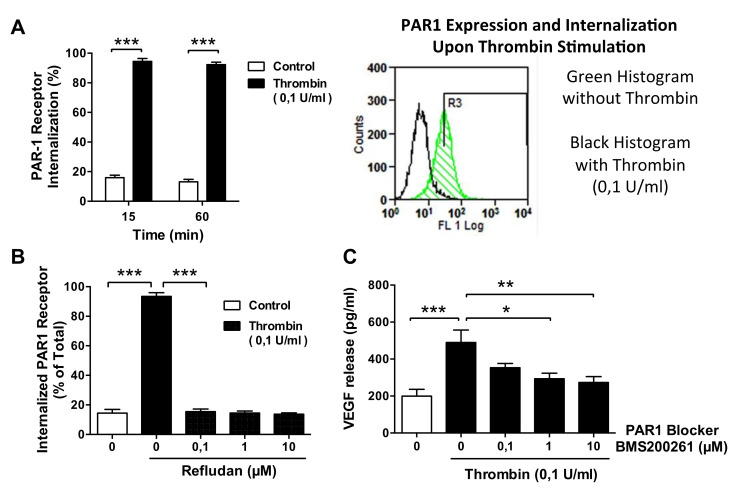
Effect of thrombin on PAR1 receptor in microvascular ECs. (**A**) Percent internalization of activated (cleaved) PAR-1 receptor by HMECs stimulated with or without thrombin (0.1 U/mL) for up to 60 min (*n* = 4) with a representative histogram of PAR-1 expression/internalization on HMECs labeled with SPAN12 antibody, stimulated for 15 min with (black) or without (green) thrombin (0.1 U/mL). (**B**) Expression of PAR-1 receptor by HMECs pretreated for 1 h with or without the thrombin inhibitor Refludan and stimulated with thrombin (0.1 U/mL) for 15 min (*n* = 4). (**C**) VEGF release in response to thrombin (0.1 pg/mL) by HMECs incubated in the presence of a PAR1 inhibitor, BMS200261, for 24 h, *n* = 10. ANOVA mean +/− SEM with * *p* < 0.05, ** *p* < 0.01, and *** *p* < 0.001.

Next, we studied changes in the cell surface expression of the PAR-1 receptor. The rate of PAR-1 receptor internalization in unstimulated control HMECs was less than 20% of the total PAR-1 expression (Figure 6A). In contrast, exposure of HMECs to thrombin (0.1 U/mL) resulted in rapid PAR-1 internalization, which reached >90% within 15 min and persisted at this level during 60 min of observation (both *p* < 0.001). Again, this effect was completely eliminated by the thrombin inhibitor Lepirudin / Refludan, and this was already evident at the lowest dose of Refludan tested (*p* < 0.001; Figure 6B). The functional role of PAR-1 for thrombin-induced VEGF production was confirmed by exposing HMECs to the PAR-1 inhibitor BMS200261. Under these conditions, the strong—more than 2-fold—induction of VEGF protein release by thrombin (*p* < 0.001) was reduced in a dose-dependent manner to the level seen in unstimulated control cells (*p* < 0.05 and *p* < 0.01; Figure 6C).

## 4. Discussion

Although the ability of thrombin to modulate the function of ECs is recognized [11], the complexity of thrombin-induced responses is still puzzling. Here, we have unraveled a part of a thrombin-signaling network by demonstrating that c-FOS is a key transcription factor controlling thrombin-induced endothelial VEGF synthesis/angiogenesis, which is in line with earlier implications of its role in angiogenesis [45,52]. This pathway connects the PAR-1 thrombin receptor, the ERK1/2 signaling cascade, and the AP-1 transcription complex. 

In this respect, our findings support and extend the previous observations [53] that thrombin at the same concentration (0.1 U/mL) induced neoangiogenesis in the chick chorioallantoic membrane and that this effect could be blocked by either the VEGF receptor inhibitor or the G-coupled protein receptor inhibitor. Indeed, both human and murine studies have shown that already very low active amounts of thrombin are physiologically active and can trigger clotting [46,51,54]. Activation of AP-1 by thrombin has been observed in ECs and linked to the thrombin-stimulated production of endothelin-1 [55], IL-8 [56], and TF [57]. 

In addition, AP-1 has been observed to contribute to VEGF induction in various cell types exposed to a number of different stimuli [45,52,58,59,60,61,62,63,64,65,66,67,68,69,70]. However, to the best of our knowledge, the involvement of AP-1 in thrombin-induced VEGF expression in the endothelium has not been reported before. Interestingly, we observed the stimulation of angiogenesis and *VEGF* expression by thrombin only at a dose of 0.1 U/mL, but not at higher concentrations (1–5 U/mL) reported previously to induce expression of dozens of genes in ECs [11]. 

Among those genes was *FLT1/VEGFR-1*, encoding the VEGF receptor, the soluble form of which (sFLT) can act as a competitor for VEGF binding. If the increase in sFLT levels induced by high thrombin was greater than that in VEGF levels, this could lead to an attenuated angiogenic response. However, while this could explain the lack of effect of high thrombin concentrations on angiogenesis, the absence of an appreciable effect on *VEGF* itself is less clear. One may hypothesize that high thrombin concentrations may induce other transcription factors that in turn interact with c-FOS to inhibit its binding to target sequences on the *VEGF* promoter [37]. 

Our study did not aim to explain whether the mechanism of angiogenesis mediated by c-FOS and VEGF represents an adaptive or maladaptive response to thrombin, as this likely depends on the pathophysiological context. On the one hand, thrombin-mediated angiogenesis may contribute to the prompt wound healing after injury. On the other hand, it may also drive abnormal angiogenesis in inflammatory pathologies; e.g., it has been observed that proinflammatory cytokines upregulate PAR-1 and that thrombin upregulates VEGF in retinal pigment epithelial cells [71]. Moreover, patients with proliferative diabetic retinopathy display an abundant expression of PAR-1 in the retinal endothelium and have high levels of thrombin and VEGF in the vitreous fluid [71].

Recently, there has been considerable interest in the potential involvement of thrombin and VEGF in COVID-19-induced ARDS. The nature of the coagulopathy and the ARDS associated with COVID-19 is not fully understood to date [39,72,73], but detailed examination of lung specimens from patients who had died from COVID-19 revealed extensive endothelial injury, microthrombosis, and neoangiogenesis [39]. 

These changes were associated with increased expression of many genes important for angiogenesis, including *VEGF.* Other studies reported on increased levels of circulating VEGF in COVID-19 [74,75,76]. The exact contribution of thrombin to increased *VEGF* expression in COVID-19 is difficult to estimate, since VEGF production may also be driven by ARDS-associated hypoxia or inflammatory cytokines, and the intensity of these stimuli may change over the course of disease [74]. 

Nevertheless, increasing evidence of endothelial damage and thrombosis in pulmonary vasculature has prompted the idea of including PAR-1 inhibitors in the treatment of COVID-19 [77]. Alternatively, the blockade of VEGF could be considered. In this respect, initial clinical observations from a limited number of patients may suggest that anti-VEGF therapy may indeed be of some benefit for patients with severe COVID-19 [78]. In mice, targeted neutralization of VEGF in the lungs was found to alleviate endotoxin-induced ARDS [79].

## 5. Conclusions

We have previously shown that c-FOS is a key transcription factor in the control of mesothelial angiogenesis in the context of peritoneal dialysis [45,52]. The induction of VEGF expression and angiogenic response by ECs in response to thrombin is a central element in vascular repair within the cardiovascular system. We here show that the exposure of HMECs to thrombin engages signaling through the PAR-1–ERK1/2–AP-1 axis to control *VEGF* transcription and secretion and concomitant VEGF-induced endothelial angiogenesis. The activity of the AP-1 transcription factor complex is regulated in a multifaceted manner [52], among others, through the availability and heterodimerization of members of the FOS and the JUN family. In addition to the constitutive engagement of c-JUN, the adaptive regulation of c-FOS described here mediates a highly dynamic responsiveness of the AP-1 transcription factor complex to environmental cues, such as thrombin, thus allowing ECs to rapidly respond to thromboinflammation. These observations offer a greater understanding of the endothelial response to thromboinflammation, which may help to interpret the results of clinical trials tackling the conditions associated with endothelial cell injury and thrombosis.

## Data Availability

Not new data.

## Supplementary Materials

The following are available online at https://www.mdpi.com/article/10.3390/cells10040910/s1, Figure S1: Supplemental Data, Table S1: Sequences of primers used in quantitative-real-time-PCR analysis, Table S2: Antibodies and reagents used for EMSA and western blot.

## Abbreviations

ARDS	Acute respiratory distress syndrome
COVID-19	Coronavirus-induced disease 2019
ECs / HMECs	Endothelial cells / Human microvascular endothelial cells
AP-1	Activator protein 1 transcription factor complex composed of subunits c-FOS and c-JUN
ERK1/2	Extracellular signal-regulated kinases 1 and 2
PAR-1	Protease activated receptor 1, thrombin receptor
TF/CD142	Tissue factor, highly procoagulant molecule triggers the TF pathway of coagulation
FI-XII / I-XIIa	Native and activated coagulation factors I-XII, respectively.
VEGF	Vascular endothelial growth factor
