# Supplementary material for: Transcriptional Regulation of Thrombin-Induced Endothelial VEGF Induction and Proangiogenic Response
Source: Cells. 2021 Apr 15;10(4):910. doi: 10.3390/cells10040910 (PMC8071415; doi:10.3390/cells10040910)
Supplement: Supplementary file 1 [file cells-10-00910-s001.zip › cells-1166138-supplementary.pdf]

Supplemental Tables and Figures

Table S1: Sequences of primers used in quantitative-real-time-PCR analysis.

Gene	Sequence Sense Primer 5′ to 3′	Sequence Antisense Primer 3′ to 5′
**VEGF**	AAGGAGGAGGGCAGAATCAT	ATCTGCATGGTGATGTTGGA
**B2M**	GTGCTCGCGCTACTCTCTCT	CGGCAGGCATACTCATCTTT
**c-FOS**	AGGAGAATCCGAAGGGAAAG	CTTCTCCTTCAGCAGGTTGG
**c-JUN**	CCCCAAGATCCTGAAACAGA	CCGTTGCTGGACTGGATTAT
**AP-1 Oligo**	CAGGCTTCACTGAGCGTCCGCAG	CAGGCACTCGAGGCCTCA GACATCTCCAGTCC

Abbreviations S1: VEGF, Vascular Endothelial Growth Factor; B2M, beta-2 microglobulin; AP-1, activator protein-1; and Oligo, oligonucleotide; c-FOS, proto-oncogene subunit of AP-1 transcription factor complex; and c-JUN, AP-1 subunit p39.

Table S2: Antibodies and reagents used for EMSA and western blot.

Antibody	Target Antigen	Host	Code	Company	Dilution
**SPAN-12 (PE-Conjugate)**	Human Thrombin Receptor (PAR-1)	Mouse	#IM2583	Beckman Coulter	1:500
**pERK1/2**	Human P44/42 MAPK	Rabbit	#9102	Cell Sig.Tech.	1:1000
**AP-1/c-FOS**	Human AP-1/c-FOS	Rabbit	#36201	Abcam	1:500
**AP-1/c-JUN**	Human AP-1/c-JUN*	Mouse	#sc-376488	SantaCruz	???
**beta-Actin**	House-keeping gene	Mouse	#A5441	Sigma	1:5000
**alpha-Tubulin**	House-keeping gene	Mouse	#T9026	Sigma	1:5000
**2nd Antibody Anti-Mouse**	Mouse antigen	Donkey	#715035150	Dianova	1:5000
**2nd Antibody Anti-Rabbit**	Rabbit antigen	Donkey	#711035152	Dianova	1:10000

Abbreviations S2: AP-1, activator protein-1; c-FOS, proto-oncogene subunit of AP-1 transcription factor complex; c-JUN, AP-1 subunit p39 (*) c-JUN antibody (B-2/sc-376488) specific for an epitope mapping between amino acids 237-273 within a highly conserved DNA binding domain of c-JUN.

Supplementary Figure S1: Supplemental Data

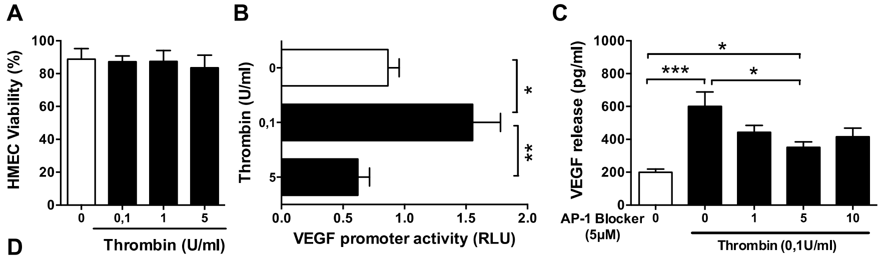

D

The VEGF promoter sequence with the c-FOS and c-JUN binding sites marked in Red or Purple, respectively: -267

GGGCGCGTGTCTCTGGACAGAGTTTCCGGGGGCGGATGGGTAATTTTCAGGCTGTGAACCTTGGTGGGGGTCGAGCTTCCCCTTCATTGCG

GCGGGCTGCGGGCCAGGCTTCACTGAGCGTCCGCAGAGCCCGGGCCCGAGCCGCGTGTGGAAGGGCTGAGGCTCGCCTGTCCCCGCCCC

CCGGGGCGGGCCGGGGGCGGGGTCCCGGCGGGGCGGAGCCATGCGCCCCCCCCTTTTTTTTTAAAAGTCGGCTGGTAGCGGGGAGG

atcgcggaggcttggggcagccgggtagctcggaggtcgtggcgctgggg +50

TCCGCAGAGC c-FOS Binding site 5′-CTGAGCGTCCGCAGAGCCCGGGCC-3′ (-131 to -154) oligo for c-FOS EMSA

TCAGGCTGTG c-JUN Binding site 5′-GTAATTTTCAGGCTGTGAACCTTG-3′ (-205 to -228) oligo for c-JUN EMSA

Figure S1 Legend: (A) HMEC viability (%) upon a 16-h incubation with different concentrations of thrombin (U/mL) as assessed with WST-8 assay [49], **(B)** Full-length VEGF promoter activity at 12 h (n=5), HMECs were transiently transfected with *VEGF* promoter constructs, stimulated with thrombin (0.1 U/mL) for 12 h and analyzed for luciferase activity. (**C**) Titration of AP-1 inhibitor SR-11302, HMECs were pre-incubated for 1 h with or without the AP-1 inhibitor SR-11302 (1, 5, and 10 μM) and then stimulated with thrombin (0.1 U/mL) for 24 h, followed by the measurement of VEGF protein release (n=6); and (D) The VEGF promter sequence with the c-FOS and c-JUN binding sites marked in red or purple, respectively, with sequences of oligonucleotides for EMSA. For easier copy-paste handling the sequences can also be found in text format below.

The VEGF promoter sequence with the c-FOS and c-JUN binding sites marked in Red or Purple, respectively: -267

GGGCGCGTGTCTCTGGACAGAGTTTCCGGGGGCGGATGGGTAATTTTCAGGCTGTGAACCTTGGTGGGGG
TCGAGCTTCCCCTTCATTGCGGCGGGCTGCGGGCCAGGCTTCACTGAGCGTCCGCAGAGCCCGGGCCCGA
GCCGCGTGTGGAAGGGCTGAGGCTCGCCTGTCCCCGCCCCCCGGGGCGGGCCGGGGGCGGGGTCCCGGC
GGGGCGGAGCCATGCGCCCCCCCCTTTTTTTTTAAAAGTCGGCTGGTAGCGGGGAGG
atcgcggaggcttggggcagccgggtagctcggaggtcgtggcgctgggg +50

TCCGCAGAGC c-FOS Binding site
TCAGGCTGTG c-JUN Binding site

5′-CTGAGCGTCCGCAGAGCCCGGGCC-3′ (-131 to -154) oligo for c-FOS EMSA
5′-GTAATTTTCAGGCTGTGAACCTTG-3′ (-205 to -228) oligo for c-JUN EMSA
